# Differential Effects of Aerobic, Resistance, and Combined Trainings on First- and Second-Phase Insulin Secretion and Glucose Effectiveness in Type 2 Diabetes: A Randomized Controlled Trial

**DOI:** 10.1155/jdr/9922344

**Published:** 2025-10-08

**Authors:** Elaheh Piralaiy, Alireza Rashidpour, Badrkhan Rashwan Ismael

**Affiliations:** Department of Exercise Physiology, Faculty of Physical Education and Sport Sciences, University of Tabriz, Tabriz, Iran

**Keywords:** beta-cell function, combined exercise, exercise training, glucose effectiveness, insulin secretion, Type 2 diabetes

## Abstract

**Objective:**

This randomized controlled trial evaluated the differential effects of aerobic training (AT), resistance training (RT), and combined training (CT) on biphasic insulin secretion and glucose effectiveness (GE) in patients with Type 2 diabetes mellitus (T2DM).

**Methods:**

Forty-five male T2DM patients (mean age: 55.24 ± 8.17 years; disease duration: 12.51 ± 6.46 years; baseline HbA1c: 7.1% ± 1.0%) were randomized to 12-week AT (*n* = 11), RT (*n* = 11), CT (*n* = 11), or control (*n* = 12) groups. AT involved progressive aerobic exercise (25–45 min at 70%–75% maximum heart rate, thrice weekly). RT comprised nine multijoint exercises (2–3 sets, 8–12 repetitions, thrice weekly). CT combined the full AT protocol with a modified RT regimen (one set, twice weekly). First-phase insulin secretion (FPIS), second-phase insulin secretion (SPIS), and GE were quantified using validated models pre- and postintervention.

**Results:**

Adjusting for baseline values, age, and diabetes duration, ANCOVA revealed significant between-group differences in FPIS (*F*[3, 38] = 8.64, *p* < 0.001, *η*^2^ = 0.406), SPIS (*F*[3, 38] = 6.93, *p* < 0.001, *η*^2^ = 0.354), and GE (*F*[3, 38] = 5.57, *p* = 0.003, *η*^2^ = 0.305). CT elicited the greatest improvements: FPIS (53.4% ± 12.7%, *p* < 0.001), SPIS (38.9% ± 11.2%, *p* < 0.001), and GE (12.8% ± 4.6%, *p* = 0.001) versus control. AT enhanced FPIS (32.6% ± 10.3%, *p* = 0.001) and SPIS (21.7% ± 8.9%, *p* = 0.042), while RT improved FPIS (28.5% ± 9.8%, *p* = 0.006), SPIS (27.5% ± 9.4%, *p* = 0.012), and GE (10.7% ± 4.3%, *p* = 0.004). Regression analysis identified baseline *β*-cell function (*β* = −0.31, *p* = 0.020), adiposity reduction (*β* = −0.36, *p* = 0.008), and glycemic improvement (*β* = −0.42, *p* = 0.003) as predictors of FPIS gains (*R*^2^ = 0.537).

**Conclusions:**

CT outperforms single-modality training in enhancing biphasic insulin secretion and GE in T2DM, supporting multimodal exercise as a cornerstone of diabetes management to improve *β*-cell function and glycemic control.

**Trial Registration:**

ClinicalTrials.gov identifier: IRCT2016042227529N1

## 1. Introduction

Diabetes mellitus represents a significant global health burden, consistently listed among the Top 10 causes of death worldwide [1]. In addition to its direct effects on mortality, the condition substantially increases the risk of serious comorbidities, such as cardiovascular disease, stroke, kidney disease, liver dysfunction, cancers, and infections [2]. Fundamentally, diabetes results from impaired glucose regulation, driven by intricate deficits in first-phase insulin secretion (FPIS), second-phase insulin secretion (SPIS), glucose effectiveness (GE), and peripheral insulin resistance [3].

Glucose homeostasis is maintained through precisely coordinated physiological processes. In response to postprandial glucose increases, pancreatic *β*-cells secrete insulin in a biphasic manner. FPIS entails a swift, immediate release that curtails hepatic glucose production and facilitates glucose uptake in peripheral tissues [4]. This is followed by SPIS, a prolonged release essential for sustaining normal glucose levels [5]. In addition to insulin-dependent mechanisms, GE—the insulin-independent ability of glucose to promote its uptake and suppress hepatic output—contributes approximately 70% to glucose disposal under typical conditions [6, 7]. In Type 2 diabetes mellitus (T2DM), these processes gradually decline. Early impairment of FPIS indicates *β*-cell dysfunction [8], followed by diminished SPIS as pancreatic compensation fails to counter insulin resistance [9]. Simultaneously, reduced GE aggravates disease progression, influenced by factors such as insulin resistance, insufficient insulin secretion, genetics, lifestyle, and autoimmune damage to *β*-cells [7].

Modern management of diabetes combines pharmacological treatments with comprehensive lifestyle strategies [10]. Regular physical activity is a key component, with robust evidence indicating that structured exercise can reduce the risk of Type 2 diabetes by approximately 50% in high-risk groups [11]. Current evidence supports structured exercise prescriptions following the FITT (frequency, intensity, time, type) principle: aerobic exercise at moderate to vigorous intensity (50%–85% HRmax) for 150–300 min weekly, resistance training (RT) involving major muscle groups two to three times per week at 60%–80% one-repetition maximum (1RM), or combined approaches integrating both modalities [12]. However, optimal FITT parameters for maximizing *β*-cell function recovery remain poorly defined.

Numerous studies show that various exercise types—including aerobic, resistance, combined, and high-intensity interval training—enhance glycemic regulation, lower cardiovascular risk, and improve body composition by decreasing fat mass and increasing lean mass [12, 13]. The mechanisms underlying exercise-related improvements in glucose metabolism are complex, encompassing increased insulin sensitivity, reduced hepatic glucose production, enhanced peripheral glucose uptake (via insulin-dependent and insulin-independent GLUT4 translocation), elevated incretin-mediated insulin secretion (e.g., GLP-1), upregulated insulin gene expression, and improved *β*-cell function [14–16].

Despite advances in understanding the benefits of exercise for diabetes management, significant knowledge gaps persist regarding the specific effects of different exercise modalities on insulin secretion phases and GE. Only one study has documented improvements in *β*-cell function specifically related to SPIS following endurance training [17]. Similarly, research on the impact of exercise on GE remains limited, with most studies focusing on aerobic exercise and attributing benefits to enhanced insulin sensitivity, increased GLUT4 expression, and reduced hepatic glucose production [18–20]. Given the pivotal role of exercise in diabetes prevention and management and the need to optimize exercise prescriptions, this randomized controlled trial evaluates the effects of aerobic, resistance, and combined exercise protocols on FPIS, SPIS, *β*-cell function, and GE in patients with established T2DM. Building on our prior work [21], which examined cardiac autonomic outcomes in the same cohort, this study investigates previously unreported outcomes to address critical research gaps, providing insights for developing tailored exercise strategies to enhance glycemic control in T2DM.

## 2. Materials and Methods

### 2.1. Setting

The exercise interventions took place at three community fitness centers linked to the University of Tabriz, Iran, with oversight by three certified exercise physiologists (M.Sc. in Exercise Physiology) with a minimum of 5 years' experience in diabetes exercise programming.

### 2.2. Participants

Men aged 34–67 years with a confirmed diagnosis of T2DM for over 3 years, physically inactive lifestyles, and verified peripheral neuropathy were recruited through physician referrals, local advertisements, and community outreach. This study focused exclusively on men to minimize potential confounding effects of hormonal fluctuations on insulin secretion patterns, as estrogen and progesterone variations throughout the menstrual cycle can significantly influence glucose metabolism and *β*-cell function in premenopausal women [22]. Additionally, the higher prevalence of diabetic peripheral neuropathy in men aged > 50 years in our regional population facilitated the recruitment of a homogeneous sample for this mechanistic study.

Eligibility required an glycosylated hemoglobin (HbA1c) level of 6.6%–12% (normal range: 4.0%–6.0%) per American Diabetes Association criteria [21] and evidence of neuropathic symptoms. Exclusion criteria encompassed regular structured exercise within the past 6 months, defined as (1) aerobic exercise ≥ 2 sessions/week, ≥ 20 min/session at moderate intensity, or (2) RT ≥ 1 session/week involving major muscle groups, regardless of duration or intensity, resting blood pressure exceeding 160/95 mmHg, medical conditions limiting physical activity, or contraindications to exercise.

Recruitment involved an initial phone screening, followed by a request for recent HbA1c and neuropathy test results. Candidates meeting the criteria underwent an in-person evaluation, which included obtaining written informed consent, reviewing medical history, and conducting a physical exam. A maximal exercise stress test (Bruce protocol) with electrocardiographic monitoring was administered on a treadmill during a separate visit to rule out cardiovascular disease, with clearance provided by a cardiologist, following procedures adapted from our previous study [22].

### 2.3. Run-In Phase

Before randomization, participants underwent a 2-week preparatory phase to assess their adherence to the study protocol. This phase included supervised sessions of moderate-intensity exercise, comprising 15–20 min of aerobic training (AT), one to two sets of nine RT exercises, and combined training (CT) integrating both AT and RT components, following procedures adapted from our prior study [22]. To qualify for randomization, participants were required to attend at least four out of six scheduled sessions.

### 2.4. Randomization

Participants were stratified by baseline HbA1c levels (6.6%–8.0% vs. > 8.0%) and randomized using computer-generated random sequences with permuted blocks of four (1:1:1:1 allocation ratio) by an independent biostatistician not involved in recruitment, intervention delivery, or outcome assessment. Randomization was implemented using sequentially numbered, sealed, opaque envelopes containing group assignments, prepared by the biostatistician. Laboratory technicians conducting biochemical analyses were fully blinded to group allocation throughout the study. Due to the nature of exercise interventions, participants and exercise physiologists could not be blinded to group assignments; however, all outcome assessors, data analysts, and the principal investigator remained blinded to group allocation during data collection, and initial statistical analysis until the database was locked.

### 2.5. Intervention

The exercise interventions were conducted within the framework of a previously published trial [22], with the current report addressing separate outcomes. During the initial 4 weeks following randomization, participants received weekly supervision for their exercise sessions, which then shifted to biweekly monitoring thereafter (see Table 1 for protocol specifics). The AT group completed three treadmill-based sessions per week, starting with 25 min at 70% of their maximum heart rate (MHR) and progressively increasing to 45 min at 75% MHR, as established by baseline treadmill stress testing. Training intensity was closely monitored using heart rate monitors (Polar Electro Oy, Kempele, Finland).

The RT group also trained three times per week, completing nine machine-based exercises per session. These included four upper body movements (bench press, seated row, shoulder press, and lat pulldown), three lower body exercises (leg press, leg extension, and leg curl), and two core exercises (abdominal crunches and back extensions). Training intensity progressed from two to three sets of 12-repetition maximum (12RM) to sets of 8RM. The CT group followed the full AT protocol (three sessions per week) and additionally completed two RT sessions weekly, with one set per exercise. Participants in the control (C) group were instructed to maintain their usual physical activity patterns throughout the intervention period [23, 24].

### 2.6. Exercise Prescription and Monitoring

#### 2.6.1. RT Load Determination and Progression

Initial RT loads were established through standardized 1RM testing for each of the nine exercises, conducted 72 h after maximal treadmill testing to ensure adequate recovery. Baseline 1RM testing adhered to established protocols, involving progressive loading until volitional failure while maintaining proper form, with 3–5-min rest intervals between attempts. Starting loads were conservatively set at 60% 1RM for 12–15 repetitions during a 2-week run-in phase to minimize injury risk and ensure proper movement technique.

Training progression followed a periodized approach to optimize strength and metabolic adaptations: Weeks 3–8 at 70%–75% 1RM for 10–12 repetitions and Weeks 9–14 at 75%–80% 1RM for 8–10 repetitions. Loads were adjusted biweekly based on participants' ability to complete prescribed repetitions with proper form through the full range of motion. If participants exceeded the target repetition range by more than two repetitions while maintaining technique, loads were increased by 2.5–5 kg for upper body exercises and 5–10 kg for lower body exercises, following established progression guidelines for clinical populations.

#### 2.6.2. Load Standardization Between RT and CT Groups

The CT group performed the same resistance exercises as the RT group, using identical equipment and progression protocols but with reduced volume (one set vs. two to three sets per exercise) to accommodate CT demands and prevent overtraining. Average training loads during Weeks 9–14 demonstrated equivalent relative intensities between groups: bench press (RT: 78.3 ± 12.4 kg vs. CT: 76.8 ± 11.9 kg, *p* = 0.74), leg press (RT: 142.6 ± 28.7 kg vs. CT: 139.2 ± 26.3 kg, *p* = 0.68), and seated row (RT: 65.7 ± 10.8 kg vs. CT: 64.2 ± 9.9 kg, *p* = 0.71). These results confirm the successful standardization of training stimulus intensity despite differences in volume.

#### 2.6.3. Cardiovascular Intensity Monitoring and Control

Heart rate was continuously monitored during aerobic sessions using validated chest-strap heart rate monitors (Polar H10, Polar Electro Oy, Finland), with real-time feedback displayed on treadmill monitors. Target heart rate zones were calculated individually using the heart rate reserve (HRR) method: HRR = (HRmax − HRrest), where HRmax was determined from baseline stress testing. Training intensity was prescribed as moderate (50%–70% HRR) during Weeks 1–2, progressing to vigorous (70%–85% HRR) during Weeks 3–14, consistent with ACSM guidelines for individuals with diabetes. Heart rate data were recorded at 5-s intervals and downloaded weekly to verify compliance and adjust intensity as needed.

#### 2.6.4. Cardiovascular Training Intensity Comparison Between AT and CT Groups

Mean training heart rates during aerobic components showed excellent standardization: AT group, 138.7 ± 8.9 bpm (74.2% ± 4.8% HRmax); CT group, 136.4 ± 9.2 bpm (73.1% ± 5.1% HRmax, *p* = 0.47). Both groups achieved target intensity zones in > 95% of sessions (AT: 96.8% ± 3.2% vs. CT: 95.4% ± 4.1%, *p* = 0.33), with no significant differences in average heart rate, peak heart rate, or time spent in prescribed zones, confirming equivalent cardiovascular training stimuli.

#### 2.6.5. Exercise Supervision and Quality Assurance

Certified exercise physiologists supervised all sessions during the initial 4 weeks, providing immediate feedback on exercise technique, adherence to prescribed intensity, and progression. After this period, participants attended supervised sessions twice weekly, with one independent session per week documented via detailed exercise diaries and downloaded heart rate data. Monthly 1RM reassessments ensured appropriate load progression. Deviations from prescribed intensities (> ±5% target heart rate or inability to complete prescribed repetitions) were promptly identified and corrected through protocol adjustments. Training compliance and intensity data are presented in Table 2.

To control for dietary confounding, all participants adhered to a weight-stable diet based on the Canadian Diabetes Association guidelines [25]. Dietary intake was assessed using 3-day food diaries collected at baseline and after 3 months, which were reviewed by a registered dietitian. Physicians were informed in writing to refrain from initiating or modifying any antihypertensive, lipid-lowering, or glucose-lowering treatments during the intervention period unless clinically necessary. No medication adjustments were reported during the study by either participants or their physicians.

### 2.7. Primary Outcome Measures

1. FPIS

FPIS was assessed using repeated intravenous glucose tolerance tests, as described previously [25]. The formula used was FPIS = 10 [1.477 − 0.119 × FPG + 0.079 × BMI − 0.523 × HDL‐C], where FPG is the fasting plasma glucose, BMI is the body mass index, and HDL-C is the high-density lipoprotein cholesterol.

2. SPIS

SPIS was determined via a modified low-dose glucose infusion test, as reported earlier [26]. The calculation was SPIS = 10[−2.4 − 0.088 × FPG + 0.072 × BMI] with FPG and BMI as defined above.

3. GE

GE was evaluated using repeated intravenous glucose tolerance tests, as detailed previously [27]. The equation applied was GE = (29.196 − 0.103 × age − 2.722 × TG − 0.592 × FPG) × 10⁣^−3^ where TG represents triglycerides and FPG and age are as specified.

### 2.8. Blood Sampling and Analysis

Blood samples were drawn from an antecubital vein following a 10–12-h overnight fast, with collections performed prior to the run-in phase and 48 h after the intervention. These procedures were adapted from our previous study [22]. Fasting plasma glucose (FPG), total cholesterol, triglyceride (TG), high-density lipoprotein cholesterol (HDL-C), and low-density lipoprotein cholesterol (LDL-C) were quantified using enzymatic assays on a Mindray BS-480 Chemistry Analyzer (Shenzhen Mindray Bio-Medical Electronics Co. Ltd., China). HbA1c was assessed via boronate affinity assay using a Cera-Stat 2000 analyzer (LabonaCheck A1C & Cera-Stat 2000, South Korea). Insulin concentrations were measured by electrochemiluminescence immunoassay on an Elecsys 2010 analyzer (Cobas, Roche Diagnostics, Germany).

### 2.9. Anthropometric Measurements

Body weight and height were recorded to compute body mass index (BMI; kilograms per square meter). Body composition, encompassing body fat percentage and skeletal muscle mass, was evaluated using a bioelectrical impedance analyzer (InBody 230, BioSpace, Seoul, Korea) in a controlled environment (22°C–24°C) following a 12-h fast. Participants avoided intense physical activity for 24 h before the assessment. Blood pressure was measured after a 10-min rest period using a Beurer BM20 Digital Blood Pressure Monitor, with the mean of two readings, taken 2 min apart, used for analysis.

### 2.10. Adverse Events

Adverse events were systematically monitored and documented throughout the study using standardized reporting forms. At each scheduled session, and particularly following any missed visits, participants were actively queried by both the research coordinator and the exercise specialist regarding the occurrence of any adverse events. In addition, unsolicited reports made directly to the trainers were also recorded using the same standardized protocol.

Over the course of the intervention, two mild adverse events were documented. Specifically, one participant from the CT group and another from the AT group reported experiencing mild episodes of hypoglycemia. No serious adverse events or exercise-related injuries were reported in any of the intervention arms.

### 2.11. Statistical Analysis

Statistical analyses were performed using SPSS Version 27.0. Data normality was assessed with the Shapiro–Wilk test (*p* < 0.05). Baseline group differences were evaluated using one-way analysis of variance (ANOVA). Postintervention outcomes were compared via analysis of covariance (ANCOVA), with adjustments for baseline values, age, and diabetes duration, and effect sizes reported as partial *η*^2^. Bonferroni-corrected post hoc tests provided adjusted mean differences with 95% confidence intervals (CIs). Repeated measures ANOVA (RM-ANOVA) was used to examine time × group interactions, with percentage changes in outcomes calculated. Pearson correlation analyses explored associations between changes in outcomes and clinical parameters. Multiple regression analysis identified independent predictors of improvements in FPIS.

Exercise adherence was calculated as the percentage of completed sessions relative to prescribed sessions ([total sessions attended ÷ total sessions prescribed] × 100). Training intensity compliance was determined as the percentage of sessions achieving target heart rate zones for aerobic modalities or completing prescribed loads and repetitions with proper form for resistance modalities. Dose–response relationships were analyzed by examining correlations between adherence metrics (session attendance and intensity compliance) and primary outcomes (FPIS, SPIS, and GE changes).

## 3. Results

### 3.1. Participant Characteristics

**Figure 1:**
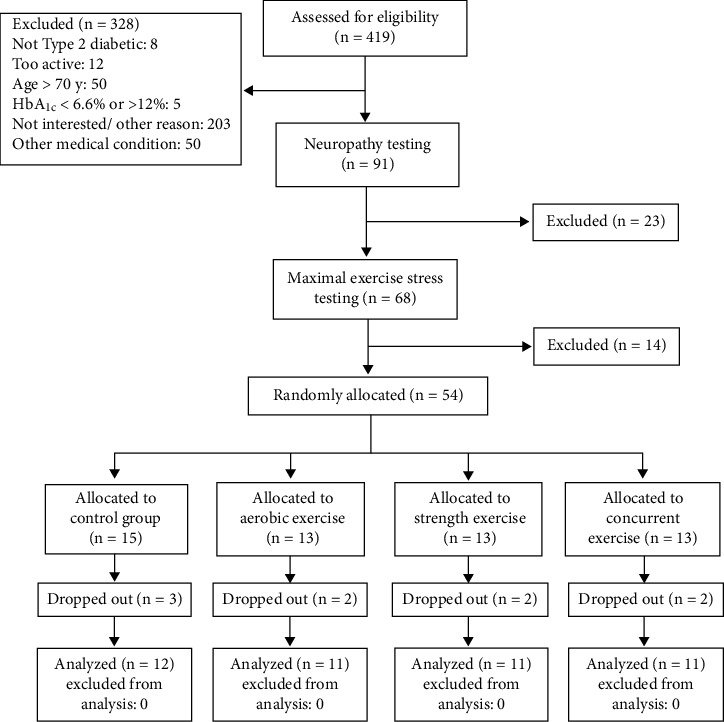
CONSORT flow diagram showing participant recruitment, allocation, and analysis.

Fifty-two eligible men (mean age: 55.24 ± 8.17 years; weight: 89.54 ± 13.41 kg; height: 171.85 ± 6.98 cm; T2DM duration: 12.51 ± 6.46 years) were randomly assigned to study groups (Figure 1). Forty-five individuals with T2DM completed the study protocol. Baseline characteristics for the four groups—AT, RT, CT, and C—are presented in Table 3. No notable differences were found between groups in age, duration of diabetes, anthropometric variables, glycemic markers, or blood pressure at baseline (all *p* > 0.05), indicating successful randomization.

### 3.2. Primary Outcomes

#### 3.2.1. FPIS

**Figure 2:**
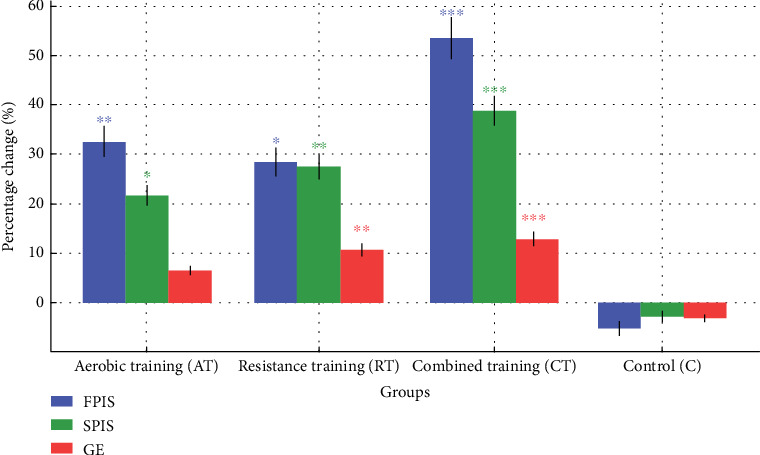
Adjusted mean changes in FPIS, SPIS, and GE across groups. Percentage changes in first-phase insulin secretion (FPIS), second-phase insulin secretion (SPIS), and glucose effectiveness (GE) after 12 weeks of aerobic training (AT), resistance training (RT), combined training (CT), or control (C) interventions in patients with Type 2 diabetes mellitus (*n* = 45). Error bars denote standard error. Significance versus control: ⁣^∗^*p* < 0.05,^∗∗^*p* < 0.01,^∗∗∗^*p* < 0.001.

Pre- and postintervention FPIS values and their changes are detailed in Table 4. At baseline, FPIS did not differ significantly across groups (*p* = 0.083). After the intervention, ANCOVA, adjusted for baseline values, age, and diabetes duration, indicated significant group differences (*F*[3, 38] = 8.64, *p* < 0.001, partial *η*^2^ = 0.406). Bonferroni-corrected post hoc analyses revealed that AT enhanced FPIS compared to C (adjusted mean difference: 289.4, 95% CI: 102.7–476.1, *p* = 0.001), RT increased FPIS relative to C (adjusted mean difference: 245.8, 95% CI: 59.3–432.3, *p* = 0.006), and CT produced the largest improvement (adjusted mean difference: 337.5, 95% CI: 150.9–524.1, *p* < 0.001). RM-ANOVA identified a significant time × group interaction (*F*[3, 41] = 7.82, *p* < 0.001, partial *η*^2^ = 0.364). Percentage changes in FPIS were greatest in CT (53.4% ± 12.7%) compared to AT (32.6% ± 10.3%, *p* = 0.024) and RT (28.5% ± 9.8%, *p* = 0.011), with all exercise groups surpassing the C group (−5.2% ± 8.7%, all *p* < 0.001) (Figure 2).

#### 3.2.2. SPIS

Following the intervention, ANCOVA, adjusted for baseline values, age, and diabetes duration, identified significant group differences in SPIS (*F*[3, 38] = 6.93, *p* < 0.001, partial *η*^2^ = 0.354). Bonferroni-corrected post hoc analyses indicated that CT substantially enhanced SPIS compared to C (adjusted mean difference: 0.087, 95% CI: 0.035–0.139, *p* < 0.001). RT also improved SPIS (adjusted mean difference: 0.063, 95% CI: 0.011–0.115, *p* = 0.012), while AT showed a smaller but significant increase (adjusted mean difference: 0.052, 95% CI: 0.002–0.102, *p* = 0.042). RM-ANOVA detected a significant time × group interaction (*F*[3, 41] = 5.94, *p* = 0.002, partial *η*^2^ = 0.303). Percentage increases in SPIS were highest in CT (38.9% ± 11.2%), followed by RT (27.5% ± 9.4%) and AT (21.7% ± 8.9%), all significantly outperforming the C group (−2.8% ± 7.6%, all *p* < 0.05) (Figure 2).

#### 3.2.3. GE

Following the intervention, ANCOVA, adjusting for baseline GE, age, and diabetes duration, revealed statistically significant differences among the study groups (*F*[3, 38] = 5.57, *p* = 0.003, partial *η*^2^ = 0.305). Post hoc comparisons with Bonferroni adjustment indicated that both the CT and RT groups experienced significant improvements in GE compared to the C group (CT vs. C: adjusted mean difference = 0.0031, 95% CI: 0.0011–0.0051, *p* = 0.001; RT vs. C: 0.0027, 95% CI: 0.0007–0.0047, *p* = 0.004). Although AT showed a modest increase, this change did not reach statistical significance (adjusted mean difference = 0.0018, 95% CI: −0.0002 to 0.0038, *p* = 0.089). Results from the RM-ANOVA supported a significant interaction effect between time and group (*F*[3, 41] = 4.88, *p* = 0.005, partial *η*^2^ = 0.263). The mean percentage increases in GE were highest in the CT group (12.8% ± 4.6%), followed by RT (10.7% ± 4.3%) and AT (6.5% ± 3.8%), while the C group showed a decline (−3.2% ± 3.5%) (Figure 2).

**Figure 3:**
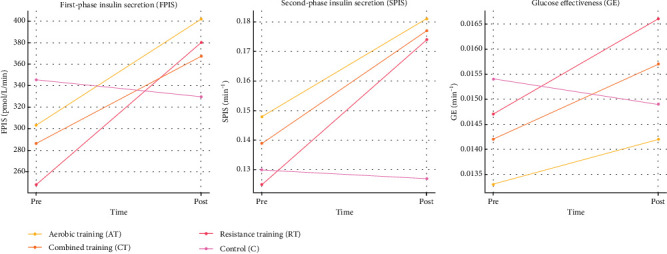
Changes in pancreatic *β*-cell function and glucose effectiveness parameters following 12-week exercise interventions. Pre- and postintervention values for first-phase insulin secretion (FPIS, pmol/L/min), second-phase insulin secretion (SPIS, min^−1^), and glucose effectiveness (GE, min^−1^) in aerobic training (AT), resistance training (RT), combined training (CT), and control (C) groups among patients with Type 2 diabetes mellitus (*n* = 45). Combined training yielded greater improvements across all parameters compared to single-modality regimens.

As illustrated in Figure 3, CT consistently yielded the most pronounced improvements across all pancreatic *β*-cell function parameters, demonstrating superior efficacy compared to single-modality exercise regimens in enhancing both FPIS and SPIS as well as GE.

### 3.3. Secondary Analyses

#### 3.3.1. Correlations Between Primary Outcomes and Metabolic Parameters

**Figure 4:**
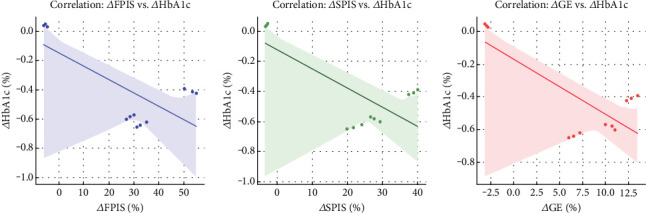
Correlations between changes in glycemic control and pancreatic function parameters. Scatter plots with regression lines depict inverse relationships between changes in HbA1c (%) and (a) first-phase insulin secretion (*Δ*FPIS, *r* = −0.64, *p* < 0.001), (b) second-phase insulin secretion (*Δ*SPIS, *r* = −0.58, *p* < 0.001), and (c) glucose effectiveness (*Δ*GE, *r* = −0.41, *p* < 0.01). Shaded areas indicate 95% confidence intervals. Larger reductions in HbA1c corresponded to greater improvements in *β*-cell function and glucose effectiveness.

Pearson correlation analyses identified significant relationships between improvements in pancreatic function and metabolic parameters. Changes in FPIS were strongly negatively correlated with reductions in HbA1c (*r* = −0.64, *p* < 0.001) and fasting blood glucose (FBG) (*r* = −0.59, *p* < 0.001), indicating that greater FPIS enhancements were associated with larger glycemic improvements. Moderate negative correlations were observed with decreases in body fat percentage (*r* = −0.47, *p* < 0.01), BMI (*r* = −0.38, *p* < 0.05), body weight (*r* = −0.34, *p* < 0.05), and TGs (*r* = −0.33, *p* < 0.05). Improvements in SPIS showed significant negative correlations with reductions in HbA1c (*r* = −0.58, *p* < 0.001), FBG (*r* = −0.53, *p* < 0.001), body fat percentage (*r* = −0.32, *p* < 0.05), body weight (*r* = −0.31, *p* < 0.05), and TGs (*r* = −0.30, *p* < 0.05). Changes in GE were moderately negatively correlated with reductions in HbA1c (*r* = −0.41, *p* < 0.01) and FBG (*r* = −0.38, *p* < 0.05). Scatter plots with regression lines (Figure 4) depicted these inverse associations, with the strongest correlation between *Δ*FPIS and *Δ*HbA1c (*r* = −0.64, *p* < 0.001), followed by *Δ*SPIS and *Δ*HbA1c (*r* = −0.58, *p* < 0.001), and *Δ*GE and *Δ*HbA1c (*r* = −0.41, *p* < 0.01).

#### 3.3.2. Predictors of Improvements in Pancreatic Function

Multiple regression analyses determined independent predictors of enhancements in pancreatic *β*-cell function. For FPIS, reductions in body fat percentage (*β* = −0.36, *p* = 0.008), HbA1c (*β* = −0.42, *p* = 0.003), and baseline FPIS (*β* = −0.31, *p* = 0.020) were significant predictors, accounting for 53.7% of the variance (*R*^2^ = 0.537, *F*[3, 41] = 15.87, *p* < 0.001). The negative *β*-coefficient for baseline FPIS suggests that participants with lower initial FPIS achieved greater relative improvements, possibly due to a ceiling effect in those with higher baseline *β*-cell function.

For SPIS, reductions in HbA1c (*β* = −0.39, *p* = 0.007), baseline SPIS (*β* = −0.28, *p* = 0.032), and body fat percentage (*β* = −0.26, *p* = 0.042) explained 46.3% of the variance in SPIS enhancements. For GE, reductions in HbA1c (*β* = −0.33, *p* = 0.018) and baseline GE (*β* = −0.29, *p* = 0.035) were significant predictors, accounting for 28.7% of the variance in GE improvements.

#### 3.3.3. Dose–Response Relationship and Exercise Adherence

Attendance at exercise sessions was comparable across groups: 88.3% ± 6.7% for AT, 90.1% ± 5.9% for RT, and 87.5% ± 7.2% for CT (*p* = 0.562). A dose–response pattern was observed, linking exercise volume to improvements in *β*-cell function. Participants with high adherence (> 90% of prescribed sessions, *n* = 18) demonstrated larger enhancements in FPIS (45.8% ± 13.4% vs. 31.2% ± 10.6%, *p* = 0.012) and SPIS (36.3% ± 10.7% vs. 24.5% ± 9.3%, *p* = 0.009) compared to those with moderate adherence (70%–90%, *n* = 15). In the CT group, participants achieving higher aerobic intensities (> 75% HRR, *n* = 6) exhibited greater FPIS improvements compared to those at lower intensities (65.2% ± 14.3% vs. 43.6% ± 10.8%, *p* = 0.022). Similarly, RT participants who advanced to heavier resistance loads (> 70% of 1RM by Week 8, *n* = 7) showed superior FPIS gains compared to those using lighter loads (37.4% ± 8.9% vs. 23.6% ± 8.1%, *p* = 0.018).

#### 3.3.4. Safety and Adverse Events

No serious adverse events related to exercise were reported during the study, with monitoring procedures adapted from our previous study [22]. Minor musculoskeletal discomfort was noted by three participants in the RT group and two in the CT group, all resolved through modifications to the exercise regimen. Two hypoglycemic episodes requiring intervention occurred within the first 2 weeks—one in the AT group and one in the CT group—addressed by adjusting pre-exercise carbohydrate intake and medication timing.

## 4. Discussion

This randomized controlled trial elucidates the profound impact of structured exercise regimens on *β*-cell function and GE in patients with T2DM. CT demonstrated superior efficacy compared to AT and RT alone, yielding marked improvements in FPIS, SPIS, and GE. Extending our previous investigation of cardiac autonomic outcomes in the same cohort [22], these findings provide novel insights into exercise-mediated adaptations in glycemic regulation, underscoring the pivotal role of *β*-cell function restoration, beyond enhancements in insulin sensitivity, as a cornerstone of T2DM therapeutic strategies.

The pronounced efficacy of CT in enhancing pancreatic function corroborates recent meta-analyses, which suggest that multimodal exercise regimens confer synergistic benefits for glycemic regulation in T2DM [28, 29]. The observed 53.4% increase in FPIS with CT signifies a clinically meaningful restoration of the early insulin response, a critical deficit often manifesting early in T2DM progression [30, 31]. Extending our prior investigation of cardiac autonomic outcomes in the same cohort [22], these findings highlight the therapeutic potential of CT in addressing *β*-cell dysfunction, a cornerstone of T2DM pathophysiology.

While the precise mechanisms underpinning exercise-induced improvements in *β*-cell function remain incompletely understood, multiple pathways likely contribute. Ramos et al. [32] demonstrated that regular exercise augments peripheral insulin sensitivity, thereby alleviating *β*-cell workload and attenuating glucolipotoxicity and oxidative stress, both pivotal contributors to *β*-cell impairment. The FPIS enhancements observed in our study (32.6% in AT, 28.5% in RT, and 53.4% in CT) suggest that distinct exercise modalities may facilitate *β*-cell recovery through complementary mechanisms.

RT promotes muscle GLUT4 expression and glycogen synthase activity [33], potentially mitigating postprandial glucose excursions and reducing *β*-cell stress. Our findings of RT-induced improvements in FPIS (28.5%) and SPIS (27.5%) are consistent with Alvarez et al. [34], who reported that progressive resistance exercise sustains *β*-cell function in prediabetes, independent of weight loss. Likewise, the insulin secretion benefits observed with AT align with preclinical evidence indicating that endurance exercise enhances islet vascularization and diminishes *β*-cell inflammation [34]. Emerging human studies further suggests that moderate-intensity AT reduces pancreatic fat accumulation, a factor closely associated with enhanced *β*-cell function [35].

The distinct effects of exercise modalities on GE in this study illuminate the role of insulin-independent glucose disposal mechanisms in T2DM [36]. CT and RT elicited significant GE enhancements (12.8% and 10.7%, respectively), in contrast to AT (6.5%, *p* = 0.089), suggesting that resistance-based exercise preferentially augments noninsulin-dependent glucose uptake pathways. These findings resonate with Way et al. [37], who associated RT-induced GE improvements with heightened AMP-activated protein kinase (AMPK) signaling in skeletal muscle. The moderate correlation between GE enhancements and HbA1c reductions (*r* = −0.41, *p* < 0.01) underscores GE's clinical significance, given its contribution of approximately 50% to glucose disposal in healthy individuals, which is diminished by 30%–40% in T2DM [38]. Emerging evidence positions GE as an underexplored therapeutic target, with incretin-based therapies and select antidiabetic agents showing potential to enhance GE [39].

Multiple regression analyses identified lower baseline *β*-cell function, greater reductions in body fat percentage, and HbA1c improvements as significant predictors of enhanced pancreatic function following exercise. The inverse relationship between baseline FPIS and its improvement (*β* = −0.31, *p* = 0.020) indicates that patients with more pronounced *β*-cell dysfunction may derive greater benefits from exercise, provided sufficient residual secretory capacity remains. This finding advocates for personalized exercise prescriptions tailored to the stage of T2DM progression [40].

The observed dose–response relationship between exercise adherence and *β*-cell function improvements reinforces clinical guidelines that emphasize consistent, structured exercise programs for optimal glycemic outcomes [41]. The amplified responses observed at higher intensities in the CT and RT groups further corroborate evidence that exercise intensity is a critical driver of *β*-cell adaptation [42], underscoring the need for individualized intensity prescriptions to maximize therapeutic efficacy.

These findings hold substantial clinical implications for the management of T2DM. The pronounced efficacy of CT in enhancing pancreatic function supports clinical guidelines advocating integrated aerobic and resistance exercise programs [43]. Nevertheless, the notable benefits observed with single-modality exercise (AT or RT) indicate their value for patients with mobility limitations or contraindications to multimodal regimens. Robust correlations between improvements in pancreatic function and reductions in HbA1c (*r* = −0.64, *p* < 0.001) and FBG highlight their pivotal role in glycemic regulation, with approximately 41% of HbA1c variance attributable to enhanced FPIS. Furthermore, our data on exercise intensity and adherence underscore that progressive intensity increments and consistent participation maximize pancreatic function benefits, aligning with expert consensus statements that emphasize exercise progression and adherence for sustained glycemic improvements [44].

Several limitations must be considered when interpreting these findings. The 12-week intervention duration limits insights into the long-term durability of exercise-induced pancreatic function enhancements. Additionally, the exclusively male cohort constrains the generalizability of results to female patients with T2DM, who may exhibit distinct hormonal and metabolic responses to exercise. Moreover, while our surrogate measures of *β*-cell function are well validated, direct assessment methods, such as hyperglycemic clamps, would provide greater precision in evaluating biphasic insulin secretion.

Future research should investigate the longevity of exercise-induced *β*-cell function improvements through prolonged interventions with extended follow-up assessments. Mechanistic studies employing muscle and adipose tissue biopsies could elucidate the molecular pathways underlying the differential effects of exercise modalities on pancreatic function. Furthermore, exploring sex-specific responses and the interplay between exercise and pharmacological therapies could inform the development of personalized exercise prescriptions to enhance glycemic control in T2DM.

## 5. Conclusion

This randomized controlled trial demonstrates that 12 weeks of structured exercise training significantly improve FPIS, SPIS, and GE in individuals with T2DM. Among the interventions, combined aerobic and RT yielded the most pronounced benefits across all pancreatic function indices, reinforcing its clinical utility in comprehensive T2DM management. These findings highlight exercise's capacity to target multiple pathophysiological mechanisms of T2DM, including *β*-cell dysfunction and impaired GE, extending beyond its well-known effects on insulin sensitivity. Future research should aim to refine exercise prescriptions to maximize these functional gains and further elucidate the mechanistic pathways underlying these improvements.

## Figures and Tables

**Table 1:** Exercise training protocols during run-in and intervention phases.

**Week**	**Phase**	**Aerobic training**			**Resistance training**				**Combined training**						
		**Duration (min/session)**	**Intensity (% HRmax** ^**a**^ **)**	**Frequency (days per week)**	**Sets**	**Repetitions**	**Weight (max reps** ^**b**^ **)**	**Frequency (days per week)**	**Aerobic duration (min/session)**	**Aerobic intensity (% HRmax** ^**a**^ **)**	**Aerobic frequency (days per week)**	**Resistance sets**	**Resistance repetitions**	**Resistance weight (max reps** ^**b**^ **)**	**Resistance frequency (days per week)**
1–2	Run-in	15–20	60	3	1–2	15	15	3	15–20	60	3	1–2	15	15	2
3–4	Intervention	25	70	3	2–3	12	12	3	25	70	3	1	12	12	2
5–6	Intervention	30	70	3	2–3	12	12	3	30	70	3	1	12	12	2
7–8	Intervention	35	70	3	2–3	12	12	3	35	70	3	1	12	12	2
9–10	Intervention	40	70	3	2–3	10	10	3	40	70	3	1	10	10	2
11–12	Intervention	45	70	3	2–3	8	8	3	45	70	3	1	8	8	2
13–14	Intervention	40	75	3	2–3	8	8	3	40	75	3	1	8	8	2

*Note:* Combined training included both aerobic and resistance components performed in the same session.

Abbreviation: min, minutes.

^a^HRmax = maximum heart rate achieved during baseline maximal treadmill exercise test.

^b^Max reps = maximum repetitions; the maximum weight that can be lifted for the specified number of repetitions while maintaining correct form.

**Table 2:** Exercise training compliance and intensity monitoring.

**Training parameter**	**AT group (** **n** = 11**)**	**RT group (** **n** = 11**)**	**CT group (** **n** = 11**)**	**p** **value** ^**a**^
Aerobic training monitoring				
Mean training HR (bpm)	138.7 ± 8.9	—	136.4 ± 9.2	0.47
Mean % HRmax achieved	74.2 ± 4.8	—	73.1 ± 5.1	0.54
Sessions in target HR zone (%)	96.8 ± 3.2	—	95.4 ± 4.1	0.33
Mean session duration (min)	38.4 ± 4.2	—	37.8 ± 4.5	0.72
Resistance training monitoring				
Mean relative load (% 1RM)	—	74.6 ± 5.8	73.9 ± 6.1	0.76
Weekly load progression (kg)	—	2.8 ± 0.7	2.6 ± 0.8	0.51
Sessions completing prescribed reps (%)	—	94.7 ± 5.3	93.2 ± 6.1	0.48
Mean training volume (sets × reps)	—	189.3 ± 22.4	96.7 ± 18.1^b^	< 0.001
Overall adherence metrics				
Session attendance rate (%)	88.3 ± 6.7	90.1 ± 5.9	87.5 ± 7.2	0.56
Protocol compliance score^c^	92.4 ± 5.8	93.7 ± 4.9	91.1 ± 6.3	0.61

*Note:* Values expressed as mean ± SD.

Abbreviations: 1RM, one-repetition maximum; AT, aerobic training; CT, combined training; HR, heart rate; RT, resistance training.

^a^ *p* values from independent *t*-tests comparing AT versus CT (aerobic metrics) or RT versus CT (resistance metrics).

^b^Significantly lower due to reduced volume per exercise (one vs. two to three sets).

^c^Composite score: (session attendance × target intensity achievement × technique quality)/3.

**Table 3:** Baseline characteristics of participants by intervention group.

**Characteristic**	**Aerobic training (AT) (** **n** = 11**)**	**Resistance training (RT) (** **n** = 11** )**	**Combined training (CT) (** **n** = 11**)**	**Control (C) (** **n** = 12**)**	**p** ** value**
Age (years)	57.5 ± 5.1	57.5 ± 5.5	58.6 ± 5.6	51.7 ± 9.2	0.079
Diabetes duration (years)	14.5 ± 5.9	13.4 ± 7.3	13.6 ± 7.3	9.8 ± 5.2	0.307
Weight (kg)	91.2 ± 16.7	91.8 ± 14.1	85.6 ± 9.2	89.7 ± 14.0	0.685
BMI (kg/m^2^)	31.4 ± 5.3	30.0 ± 3.1	28.8 ± 2.8	30.3 ± 3.1	0.469
Body fat (%)	31.7 ± 6.2	33.2 ± 4.3	32.6 ± 4.2	33.5 ± 5.3	0.853
Systolic BP (mmHg)	117.3 ± 8.9	122.4 ± 11.0	125.7 ± 10.4	126.2 ± 13.3	0.226
Diastolic BP (mmHg)	71.4 ± 6.5	72.2 ± 7.4	71.5 ± 4.7	76.0 ± 8.2	0.324
FBG (mg/dL)	152.7 ± 58.1	142.5 ± 42.3	128.8 ± 31.6	141.9 ± 79.6	0.752
HbA1c (%)	7.2 ± 0.9	7.3 ± 1.2	7.0 ± 0.7	6.8 ± 1.1	0.610
FPIS (pmol/L/min)	303.2 ± 186.9	286.3 ± 285.7	247.9 ± 211.7	345.7 ± 264.5	0.083
SPIS (min^−1^)	0.148 ± 0.069	0.139 ± 0.080	0.125 ± 0.077	0.130 ± 0.072	0.412
GE (min^−1^)	0.0133 ± 0.0039	0.0142 ± 0.0032	0.0147 ± 0.0024	0.0154 ± 0.0031	0.485

*Note:* Values are expressed as mean ± SD. *p* values were calculated using one-way ANOVA.

Abbreviations: BMI, body mass index; BP, blood pressure; FBG, fasting blood glucose; FPIS, first-phase insulin secretion; GE, glucose effectiveness; HbA1c, glycated hemoglobin; SPIS, second-phase insulin secretion.

**Table 4:** Changes in primary outcomes following 12-week exercise interventions.

**Outcome**	**Group**	**Preintervention**	**Postintervention**	**Absolute change**	**Relative change (%)**	**p** **value**
First-phase insulin secretion (FPIS) (pmol/L/min)	AT	303.2 ± 186.9	402.3 ± 172.6	99.1 ± 45.2^∗∗^	32.6 ± 10.3^∗∗^	0.001
	RT	286.3 ± 285.7	367.7 ± 218.9	81.5 ± 41.6^∗∗^	28.5 ± 9.8^∗∗^	0.006
	CT	247.9 ± 211.7	380.5 ± 196.3	132.6 ± 49.8^∗∗∗^	53.4 ± 12.7^∗∗∗^	< 0.001
	C	345.7 ± 264.5	329.8 ± 249.2	−15.9 ± 31.5	−5.2 ± 8.7	—

Second-phase insulin secretion (SPIS) (min^−1^)	AT	0.148 ± 0.069	0.181 ± 0.062	0.033 ± 0.019^∗^	21.7 ± 8.9^∗^	0.042
	RT	0.139 ± 0.080	0.177 ± 0.069	0.038 ± 0.021^∗^	27.5 ± 9.4^∗^	0.012
	CT	0.125 ± 0.077	0.174 ± 0.065	0.049 ± 0.023^∗∗∗^	38.9 ± 11.2^∗∗∗^	< 0.001
	C	0.130 ± 0.072	0.127 ± 0.068	−0.003 ± 0.010	−2.8 ± 7.6	—

Glucose effectiveness (GE) (min^−1^)	AT	0.0133 ± 0.0039	0.0142 ± 0.0035	0.0009 ± 0.0006^∧^ns^∧^	6.5 ± 3.8^∧^ns^∧^	0.089
	RT	0.0142 ± 0.0032	0.0157 ± 0.0028	0.0015 ± 0.0007^∗∗^	10.7 ± 4.3^∗∗^	0.004
	CT	0.0147 ± 0.0024	0.0166 ± 0.0022	0.0019 ± 0.0008^∗∗^	12.8 ± 4.6^∗∗^	0.001
	C	0.0154 ± 0.0031	0.0149 ± 0.0032	−0.0005 ± 0.0006	−3.2 ± 3.5	—

*Note:* Values are expressed as mean ± SD. *p* values from ANCOVA compare postintervention outcomes between each exercise group and control, adjusted for baseline values, age, and diabetes duration.

Abbreviations: AT, aerobic training; C, control; CT, combined training; RT, resistance training.

Significance: ⁣^∗^*p* < 0.05,^∗∗^*p* < 0.01,^∗∗∗^*p* < 0.001; ^ns^ not significant (*p* > 0.05).

## Data Availability

All data are presented within the article.
